# The Combination of Presurgical Cortical Gray Matter Volumetry and Cerebral Perfusion Improves the Efficacy of Predicting Postoperative Cognitive Impairment of Elderly Patients

**DOI:** 10.3390/tomography10090104

**Published:** 2024-09-01

**Authors:** Weijian Zhou, Binbin Zhu, Yifei Weng, Chunqu Chen, Jiajing Ni, Wenqi Shen, Wenting Lan, Jianhua Wang

**Affiliations:** 1 Department of Radiology, The First Affiliated Hospital of Ningbo University, Ningbo 315010, China; zwj19960105@126.com (W.Z.); zz303406191@163.com (J.N.); shenwenqi07@163.com (W.S.); fyylanwenting@nbu.edu.cn (W.L.); 2 Department of Anaesthesiology, The First Affiliated Hospital of Ningbo University, Ningbo 315010, China; fyzhubinbin@nbu.edu.cn; 3 Health Science Centre, Ningbo University, Ningbo 315211, China; 17815969498@163.com; 4 Department of Radiology, The First Affiliated Hospital of Xiamen University, 55 Zhenhai Road, Siming District, Xiamen 361026, China; fmriwengyf@126.com

**Keywords:** postoperative cognitive dysfunction, arterial spin labelling, cerebral blood flow, cortical segmentation, cortical grey matter

## Abstract

Background: Postoperative cognitive dysfunction (POCD) is a common complication of the central nervous system in elderly surgical patients. Structural MRI and arterial spin labelling (ASL) techniques found that the grey matter volume and cerebral perfusion in some specific brain areas are associated with the occurrence of POCD, but the results are inconsistent, and the predictive accuracy is low. We hypothesised that the combination of cortical grey matter volumetry and cerebral blood flow yield higher accuracy than either of the methods in discriminating the elderly individuals who are susceptible to POCD after abdominal surgery. Materials and Methods: Participants underwent neuropsychological testing before and after surgery. Postoperative cognitive dysfunction (POCD) was defined as a decrease in cognitive score of at least 20%. ASL-MRI and T1-weighted imaging were performed before surgery. We compared differences in cerebral blood flow (CBF) and cortical grey matter characteristics between POCD and non-POCD patients and generated receiver operating characteristic curves. Results: Out of 51 patients, 9 (17%) were diagnosed with POCD. CBF in the inferior frontal gyrus was lower in the POCD group compared to the non-POCD group (*p* < 0.001), and the volume of cortical grey matter in the anterior cingulate gyrus was higher in the POCD group (*p* < 0.001). The highest AUC value was 0.973. Conclusions: The combination of cortical grey matter volumetry and cerebral perfusion based on ASL-MRI has improved efficacy in the early warning of POCD to elderly abdominal surgical patients.

## 1. Introduction

Postoperative cognitive dysfunction (POCD), as a common perioperative complication of the central nervous system, primarily affects memory, attention and executive skills in elderly cohorts after surgery [1,2]. In Western countries, the prevalence of POCD is up to 38% [1,2], but it is only 13.1% in China, according to a cohort study [3]. However, this does not mean that the actual incidence of POCD in China is lower than that in Europe and the United States. To a large extent, the awareness of POCD in China, both among medical staff and the public, is significantly lower than that in Western countries, so the awareness of perioperative cognitive function assessment, screening and monitoring of patients is obviously insufficient, resulting in a significantly higher rate of missed diagnoses of POCD than that in developed countries. POCD can lead to a lot of problems such as prolonging the duration in hospital, increasing the cost of postoperative care, affecting the quality of life [4] and even increasing the postoperative mortality within one year [5].

Nowadays, the diagnostic method of POCD is to compare preoperative and postoperative cognition using neuropsychological tests and clinical assessments [6]. Many neuropsychological tests are used to detect POCD, such as Mini-Mental State Examination (MMSE), Montreal Cognitive Assessment (MoCA), Hopkins Verbal Learning Test (HVLT), Trail Making Test A and B (TMT A and B), Stroop Colour and Word test (SCWT) and so on [7]. However, there are some disadvantages of neuropsychological tests. Firstly, it is hard for patients to complete neuropsychological tests after surgery because of surgical trauma, anaesthesia or medications [6]. Secondly, apart from the usual quick bedside tests, such as MMSE and MoCA, we often need some other validated neuropsychological tests in addition, which often results in a heavy workload and difficult patient cooperation [8]. During the assessment process, due to the learning effect, some patients will respond according to their previous experience during the postoperative evaluation, which leads to the evaluation results being better than their actual situation [9].

Anaesthesia and surgery can affect cognition in many ways. Firstly, inflammation plays a vital role in conducting the occurrence of POCD; surgical trauma can cause a release of danger-associated molecular patterns (DAMPs), including high-mobility group 1 (HMGB1) and S100A8, and lead to a pro-inflammatory cascade, which contributes to the inflammation of the nervous system and disruption of the blood–brain barrier (BBB) [10,11]. Secondly, cerebral perfusion fluctuations are very common during major surgery, and the brain can keep a stable cerebral perfusion with cerebrovascular autoregulation [12]. When the mean arterial pressure (MAP) is higher or lower than the limit of the autoregulation zone, or the cerebrovascular autoregulation is impaired, it can lead to injuries of the brain [13,14]. Thirdly, microemboli can obstruct some smaller arteries, arterioles and capillaries during a surgical operation, such as cross-clamping or canulation/decannulation, or from a cardiotomy suction [7], which contributes to POCD. Additionally, the neurotoxicity of volatile anaesthetics also contributes to the occurrence of POCD; volatile anaesthesia can change the morphology of neurons, increase caspase-3 expression and culminate in neuronal apoptosis [7,15]. Another hypothesis is that POCD may reflect a kind of acceleration of Alzheimer’s disease (AD) or cerebrovascular disease pathology [4,16,17,18]. Anne et al. discovered that the atrophy of AD-related neocortical regions [19], including the medial temporal lobe, temporal pole, inferior temporal pole and apical pole [20], is linked to long-term cognitive decline at 36 months after surgery.

Based on structural MRI, the volume of grey matter in some brain areas were found to be associated with POCD. POCD commonly appears as a decline in memory and executive abilities. These mental tasks are chiefly managed by high-level cortical regions, including areas like the prefrontal cortex and temporal lobes, as well as the thalamus. These brain areas collaborate to handle sophisticated cognitive functions; the impairment of them may lead to POCD [21]. Maekawa et al. found that POCD patients have lower pre-existing volumes of bilateral hippocampal and entorhinal cortex [22]. Johnathon and his colleagues reported that patients with POCD showed a smaller volume of the thalamus [23]. Sato et al. conducted a study of breast cancer chemotherapy patients and found that breast cancer chemotherapy patients had poor performance in attention, measured by the Dual-Choice Auditory Task experiment (D-CAT), which was significantly correlated with the patients’ postoperative thalamic volume [24]. However, in some conditions, the outcomes might not be consistent across studies. For example, the study by Marinus et al. did not find the same result about the thalamus [25]. And there are also contradictions in Maekawa’s [22] and Price’s [26] results on preoperative hippocampal and entorhinal cortex volumes. The lack of uniform neuropsychological test batteries between these studies may be a possible reason for those inconsistent results.

Kant et al. expounded that the correlation between cerebrovascular disease and POCD is greater in terms of incidence rate compared with neurodegenerative diseases [27]. Arterial spin labelling (ASL) is a non-invasive technique that measures cerebral blood flow (CBF) by magnetically tagging the body’s own arterial water to serve as a tracer. This labelled water subsequently diffuses into the brain tissue, allowing for the effective measurement of blood flow [28], which is an advanced technology with the advantages of non-invasiveness and absence of exposure to ionising radiation [29]. However, there are few scholars that have used this tech to detect POCD. Only one study performed by Xue et al. found that their POCD group showed lower CBF in several specific brain regions and higher CBF in the triangularis of the inferior frontal gyrus compared to the non-POCD group (*p* < 0.001) [30]. And, Tammy and colleagues discovered a positive correlation between CBF in the posterior cingulate and praecuneus regions and the performance of language functions, as well as attention [31]. Lin et al. found that postoperative cognitive improvements in patients who underwent carotid endarterectomy based on MMSE and MoCA were associated with cerebral perfusion normalisation, measured by ASL-MRI [32]. The studies of detection and prediction of POCD with ASL are few, so it is hard to judge the efficacy of ASL in the diagnosis and prediction of POCD.

Another advantage of MRI is multiparametric imaging. Single-modal MR imaging has various disadvantages. Structural MRI alone just provides differences in morphology, and is short on information on the function and metabolic activity of tissues; however, the change in morphology is a long process and it may not be conducive to early diagnosis [33,34]. And ASL imaging has difficulties in assessing subtle changes in brain structure because the spatial and temporal resolution is limited and there is a lack of a suite of uniform parameters to perform this examination. Another barrier of ASL in clinical practice is its low signal-to-noise ratio, which leads to lower image quality [35,36,37]. Henry et al. have performed a study to combine the parameters of CBF in posterior cingulate gyri and hippocampal volumes to detect Alzheimer’s disease in cognitively normal elderly adults. The highest area-under-curve value was 0.944, by combining cerebral blood flow at the middle cingulate gyrus and normalised right and left hippocampal volumes [38]. However, there is still no study which combinates multiple parameters to predict the occurrence of POCD at present. Moreover, many previous experiments have not evaluated its diagnostic and early-warning efficacy.

In this research, our goal is to identify the differences between structural-MRI cortical volumetry and CBF, as measured by ASL-MRI. We also aim to assess how effective cortical grey matter volumetry, ASL-MRI-derived CBF and their combination are in distinguishing early POCD in elderly patients undergoing abdominal surgery. Pre-surgical MR examination can provide baseline information on patients in order to find early predictors of POCD; in addition, postoperative MRI may be affected by surgery and anaesthesia in the short term and does not reflect the long-term cognitive status of the patient. From the aspect of clinical practicability, preoperative MRI is easier to obtain and less intrusive to the patient. We hypothesise that combining preoperative structural-MRI cortical grey matter volumetry with preoperative ASL-MRI perfusion could be more effective in diagnosis than using each method individually.

## 2. Materials and Methods

### 2.1. Study Population

All patients collected in this current study are those scheduled for laparoscopic surgery through gastrointestinal surgery, anorectal surgery, hepatobiliary surgery and urology from September 2021 to January 2024. This investigation was approved by the Ethics Committee of the First Affiliated Hospital of Ningbo University (approval number: 107A-02). We obtained informed consent from every participant after a full protocol explanation. The principles of the Declaration of Helsinki were adhered to by the authors.

Eligibility criteria were as follows: (1) age ≥ 60 years; (2) patients undergoing laparoscopic or open abdominal surgery (surgery duration > 2 h); (3) non-illiterate patients (education level ≥ 1 year); (4) patients without colour blindness, visual impairment or hearing impairment; (5) informed consent from participants.

Exclusion criteria were as follows: (1) left-handed or ambidextrous because of functional differences between the left and right hemispheres, which may influence the findings; (2) pre-existing cognitive impairment diseases (such as Alzheimer’s disease, Parkinson’s disease, etc.) in order to eliminate the confounding factors of preoperative cognitive dysfunction as much as possible; (3) pre-existing psychiatric disorders; (4) patients with severe depression or anxiety, because it can affect the result of the neuropsychological tests (NPTs) and it may be hard for them to cooperate with us; (5) pre-existing neurological diseases that could cause cognitive impairment, such as extensive cerebral infarction, encephalitis, brain tumours, traumatic brain injury, etc.; (6) other systemic diseases that could cause cognitive impairment, such as neurosyphilis, HIV/AIDS, severe anaemia, vitamin B12 or folate deficiency, hepatic encephalopathy, carbon monoxide poisoning, etc.; (7) history of drug or alcohol abuse; (8) contraindications for MRI; (9) poor quality of MR images (e.g., artifacts, incomplete scans); (10) inability to cooperate to complete neuropsychological assessment scales.

### 2.2. Study Processes

**Figure 1:**
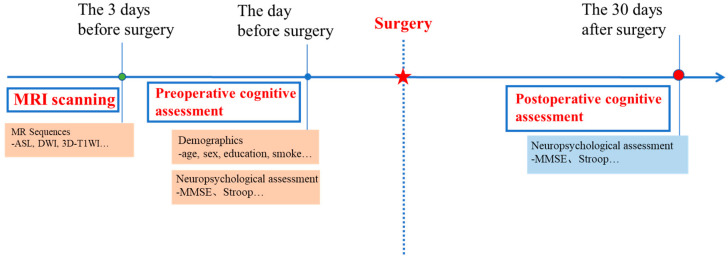
Experimental workflow.

The experimental workflow is shown in Figure 1. All patients underwent pre- and postoperative cognitive assessment and preoperative MRI scanning 1 to 3 days before the abdominal surgery. An experienced radiologist evaluated the MR image quality and excluded those with pre-existing neurological diseases. The baseline neuropsychological tests (NPTs) would be performed one day before the surgery. The second NPTs would be performed 30 days postoperatively.

Neuropsychological evaluation was conducted by the same well-trained neuropsychologist at two time points for patients. In addition, subjects were first screened to exclude subjects with pre-exiting cognitive impairment. Patients who had MCI, AD or PD and other diseases affecting cognition were not tested any further.

### 2.3. Neuropsychological Evaluation and POCD Definition

The test battery consisted of 6 tests: a Clock-Drawing Test (CDT) to measure visuospatial and executive function, which are associated with the parietal and temporal lobe [39]; a verbal fluency test (VFT) to measure the language function and attention, which are associated with the frontal and temporal lobe [40]; the Trail Making Test A and B (TMT A and B) for assessing executive function, which is associated with frontal lobe abilities [41]; a Forward and Backward Digit Span Test (DST forward and backward) to assess memory function and attention, which are associated with the anterior cingulate gyrus and inferior temporal gyrus; a Stroop Colour and Word Test (SCWT), which is a test of attention that can activate the anterior cingulate, insula, premotor and inferior frontal regions [42]; and a Mini-Mental State Examination (MMSE) for a global cognition assessment which includes 6 cognitive domains: memory, orientation, registration, attention, language and visual construction ability [43]. In addition, we used the Instrumental Activities of Daily Living (IADL) Scale and Pittsburgh Sleep Quality Index (PSQI) to assess the patients’ ability to live independently and the status of their sleeping. Different versions of the consent scale were adopted preoperatively and postoperatively to eliminate the effect of the patient’s learning.

The criteria of cognitive decline were applied to the test results [44,45]:

1. If the score of a certification test after surgery is reduced by 20% compared with the score before surgery, it can be judged that the cognitive function of this dimension has declined;
2. POCD can be diagnosed if the cognitive score of one or more postoperative items decreases by 20% or more compared with the preoperative level.

### 2.4. MRI Scan

All participants underwent MRI scans using three 1.5 T MRI machines (GE Healthcare, Chicago, IL, USA; Siemens, Munich, Germany; United Imaging, Shanghai, China), each outfitted with a 32-channel head coil. For volumetric studies, a whole-brain T1-weighted 3D magnetisation-prepared rapid gradient echo (MPRAGE) was executed, featuring a TR/TE of 9.7/10 ms, a flip angle of 13° and a slice thickness of 1 mm. The field of view was set to 240 mm × 240 mm, with a 256 × 256 matrix size, resulting in a voxel size of 0.9375 mm × 0.9375 mm × 1 mm. In total, 140 slices were acquired over a 5 min scan duration. Additionally, a 3D pseudocontinuous arterial spin-labelling (3D-pCASL) procedure, with background suppression, was conducted to measure whole-brain cerebral blood flow (CBF). This included a TR/TE of 4500/10 ms, flip angle of 180° and slice thickness of 4 mm across a 240 mm × 240 mm field of view and a 256 × 256 matrix, producing a voxel size of 0.9375 mm × 0.9375 mm × 1 mm with 32 slices, NEX of 2, 1500 ms labelling time and 1800 ms post-labelling delay. The total scan lasted 4 min and 30 s.

Participants were instructed to remain awake, relax with their eyes closed and keep their heads still during the scans. They underwent standard clinical screening for MRI safety and potential contraindications beforehand.

During the image collection, all scans were checked for artifacts to ensure quality. While other sequences such as fluid-attenuated inversion recovery, three-dimensional T2 and diffusion imaging were also collected, they were not included in the current analysis.

### 2.5. MRI Analysis

#### 2.5.1. ASL Perfusion Image

**Figure 2:**
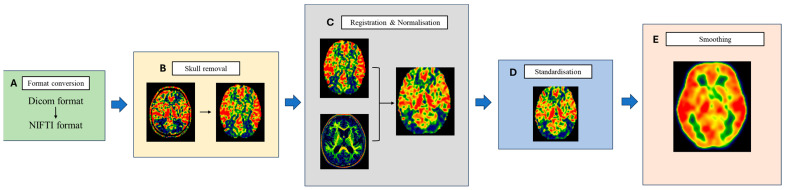
ASL-MRI processing streamline. (**A**) Image format conversion: all the imaging of patients are converted from Dicom format to NIFTI format. (**B**) Skull removal: removing the extracerebral parts of the CBF images. (**C**) Registering the CBF map onto the T1 images and normalising them to the Montreal Neurological Institute (MNI) template. (**D**) Standardisation of CBF values of the whole brain with the mean division method. (**E**) Spatially smoothing of CBF maps: spatial-filtering techniques are applied to smooth the feature map to eliminate local noise while maintaining the global trend.

The ASL-MRI processing streamline is shown in Figure 2. Firstly, the 3D-pCASL image was converted to the Neuroimaging Informatics Technology Initiative (NIFTI) format, then, the extracerebral tissue of all CBF images was removed using the *Swiss Skull Stripper* module of the 3D slicer software, which could provide a success rate of registration at the beginning. The later preprocessing and statistical analyses were performed with SPM12 (statistical parametric mapping software, Wellcome Trust Centre for Neuroimaging, London, UK), implemented in MATLAB 2021 (Mathworks, Natick, MA, USA). The CBF maps were registered onto structural T1-weighted images and then normalised onto the reference image: a Montreal Neurological Institute (MNI) template. After normalisation, the CBF values of the whole brain were standardised using the *Mean Division* method in DPABI to eliminate individual differences. The CBF maps were then spatially smoothed with an isotropic 8 mm full width at half-maximum Gaussian filter.

#### 2.5.2. High-Resolution 3D-T1WI Image

**Figure 3:**
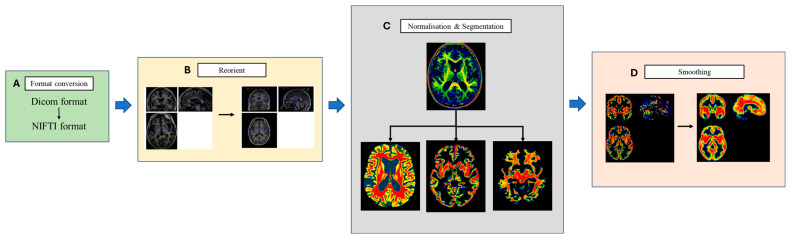
Cortical segmentation streamline. (**A**) Image format conversion: All the images of patients are converted from Dicom format to NIFTI format. (**B**) Reorientation of structural imaging into a standard coordinate space. (**C**) Normalising the T1 images to the Montreal Neurological Institute (MNI) template, then separating them into three parts including grey matter, white matter and whole brain. (**D**) Spatial smoothing of cortical grey matter maps: spatial-filtering techniques are applied to smooth the feature map to eliminate local noise while maintaining the global trend.

The processing of cortical segmentation is shown in Figure 3. Firstly, the structural T1 image were converted to the Neuroimaging Informatics Technology Initiative (NIFTI) format. Next, using the “Display” function in the SPM12 software, the images were imported and positional correction performed by placing the original structural image data in a standard coordinate space. Then, all the 3D-T1WI were processed with the automated procedures in the Computational Anatomy Toolbox (CAT12) with SPM12 based on the MATLAB 2021 platform (MathWorks, Natick, MA, USA). The workflow for segmentation in CAT12 encompasses initial voxel-based processing followed by surface-based processing. During the voxel-based stage, 3D-T1-weighted images underwent denoising, bias-field correction and skull stripping. After correcting local intensities, the images were segmented into grey matter and white matter, as referenced in [46]. These tissue segments were then aligned to the MNI standard space through spatial normalisation. Finally, the grey matter (GM) maps were enhanced using an isotropic Gaussian filter with an 8 mm full width at half maximum for spatial smoothing.

**Figure 4:**
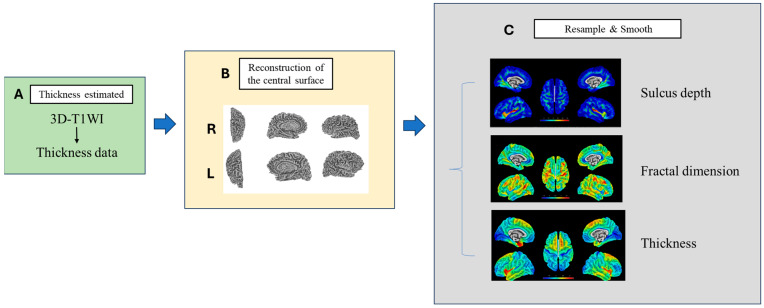
Surface morphological construction processing, image preprocessing and anatomical structure segmentation have been completed in the previous step. (**A**) Thickness estimate of cortical grey matter: Anatomical models are used to estimate the thickness of the cortex, and the method for thickness measurement is based on distance transformation. (**B**) Reconstruction of brain surface: The outer and inner surfaces of the brain are extracted from the segmented anatomical structures, topological correction and 3D modelling are performed and the reconstructed surface structure is visualised. (**C**) Resample and Smoothing of map of “Sulcus depth”, “Fractal dimension” and “Thickness”: The initial values of each feature (such as gyral depth, fractal dimension and thickness) are calculated and a map is generated, then initial data are resampled into standard space and spatial filtering techniques are applied to smooth the feature map to eliminate local noise while maintaining the global trend.

After the voxel-based processing, the surface-based processing was performed. The process of 3D morphological construction of the brain surface is shown in Figure 4. The estimation of cortical thickness and the reconstruction of the central surface were carried out simultaneously through the utilisation of a projection-based thickness method in a single step [47]. Following the initial surface reconstruction, topological imperfections were rectified utilising spherical harmonics [48]. This was succeeded by a surface enhancement process, leading to the ultimate formation of the central surface mesh. Subsequent to this, the individual central surfaces were aligned to the Freesurfer ‘FsAverage’ template through spherical mapping with minimal distortions [49]. Then the surface structures were resampled and smoothened to produce the surface maps of “Sulcus depth”, “Fractal dimension” and “Thickness”.

### 2.6. Statistical Analysis

Using SPSS26 software for the statistical analysis of demographic data and cognitive scores, all continuous variables are presented in the form of “median, interquartile range”. For normally distributed data, a two-sample *t*-test is used, while for skewed data, the Mann–Whitney U test is employed. Categorical variables are presented in the form of “count, percentage”, and statistical analysis is conducted using the Chi-square test. The aim is to explore the differences in demographic characteristics and preoperative cognitive performance between POCD patients and NPOCD patients.

Using SPM12 software, the voxel-wise two-sided *t*-test was carried out to compare cerebral perfusion across the whole brain as well as the grey matter’s volume, cortical thickness, fractal dimension, or sulcus depth between the POCD and NPOCD groups. For the VBM analysis, total intracranial volume was included as a covariate since SBM analysis does not typically scale with brain size. Significance was established with a *p*-value of <0.05, applying cluster-level family-wise error correction for multiple comparisons, or at an uncorrected *p*-value of <0.001 to identify subtle atrophic changes [50].

After comparing the abnormal-perfusion brain regions obtained from the voxel-based imaging statistical analysis between the POCD and non-POCD groups, DPABI 4.3 software was used to create a mask from these regions as regions of interest (ROIs). The local CBF values and grey matter volume for each subject was extracted using the mask as the ROI. Then, in SPSS26, binary logistic regression was performed on the CBF values and grey matter volume of the brain regions obtained. A receiver operating characteristic curve (ROC curve) was generated and the area under the curve (AUC) was calculated to assess the diagnostic performance of these imaging features in distinguishing between POCD and non-POCD individuals.

## 3. Results

### 3.1. Participants and Clinical Data

**Figure 5:**
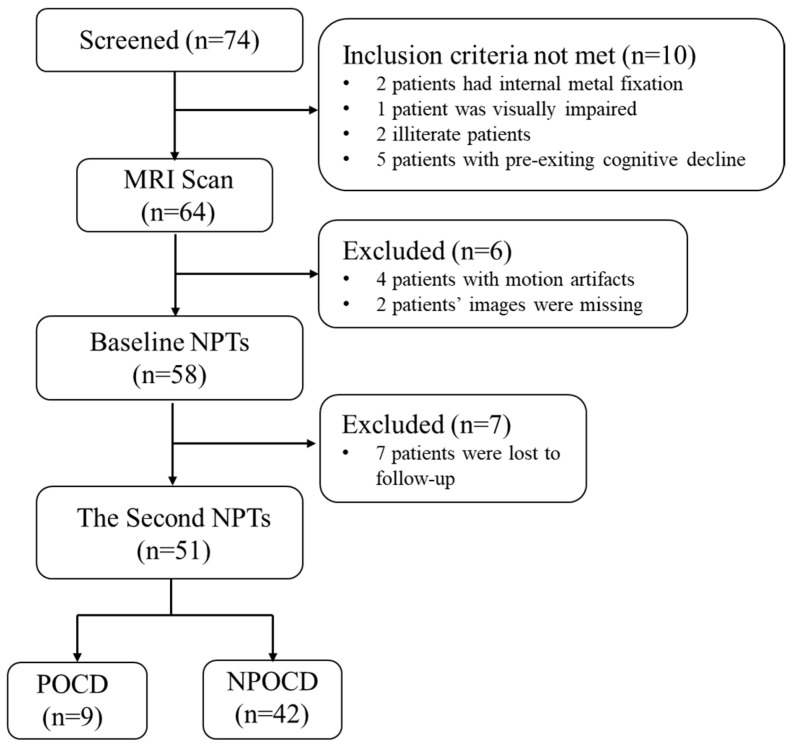
Flow diagram for study assessments. NPTs, neuropsychological tests; MRI, magnetic resonance imaging; POCD, postoperative cognitive dysfunction; NPOCD, non-POCD.

The study included 74 patients, as detailed in Figure 5. Of these, 51 patients completed both the preoperative MRI scans and neuropsychological tests at two timepoints. Within this group, there were 15 females and 36 males. Postoperative cognitive dysfunction (POCD) was identified in 9 out of the 51 patients (17.65%) by the 30th day following surgery.

The differences in demographic characteristics between the POCD and non-POCD groups are shown in Table 1. Firstly, in terms of educational years, those of the POCD-group patients were significantly lower than in the NPOCD group (*p* < 0.05). According to the Pittsburgh Sleep Quality Index scores reflecting the quality of sleep in the past month, the POCD-group patients’ scores were significantly higher than those of the NPOCD group (*p* < 0.05), indicating poorer sleep quality in the POCD group compared to the non-POCD group. Additionally, based on the Instrumental Activities of Daily Living (IADL) scale scores reflecting the patients’ daily life abilities, this study found that the daily life abilities of the POCD patients were weaker than those of non-POCD patients. Interestingly, the daily exercise time of POCD patients was significantly higher than that of non-POCD patients.

The neuropsychological test scores are showed in Table 2 for both baseline and 30 days after surgery. The preoperative scores of the VFT of the POCD patients were significantly lower than those of the NPOCD group (*p* < 0.05), but the preoperative scores of the other NPTS were similar between the two groups (*p* > 0.05).

In the postoperative cognitive assessment results, the POCD group performed significantly worse than the NPOCD group in the MMSE, VFT and TMT B tests (*p* < 0.05). This indicates that, in our study, patients with POCD primarily exhibited major impairments in language and executive functions, which aligns with clinical presentations reported in the literature [51].

### 3.2. Voxel-Based Analyses of CBF Maps Based on ASL

**Figure 6:**
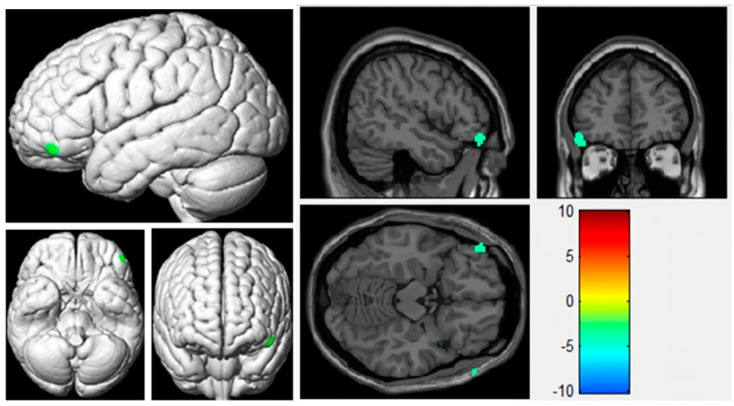
Differences in cerebral blood flow (CBF) between the POCD and NPOCD groups. Voxel-wise comparison of the partial volume-corrected ASL revealed decreased CBF in the left inferior frontal gyrus. The voxel-based comparison results are overlain on the Montreal Neurologic Institute standard brain images in the coronal, axial and sagittal planes. Due to the correction for multiple comparisons resulting in a large cluster of brain regions, only selected sections showing brain regions with statistical differences are displayed. No significant differences were observed in the overlapping brain regions. The colour gradient from blue to red represents varying levels of blood flow, with blue indicating low flow and red indicating high flow.

Among the 51 patients, a total of 42 sets of CBF images were collected, with 9 sets belonging to POCD patients and 33 sets belonging to non-POCD patients. A two-sample *t*-test comparing perfusion differences between the two groups of subjects showed statistical significance. The brain regions with significantly decreased perfusion were mainly located in the left inferior frontal gyrus. According to Brodmann Area (BA) partitioning, the regions with decreased perfusion were mainly in BA47 (left inferior frontal gyrus), which included 14 voxels. Compared to the non-POCD group, patients in the POCD group showed significant perfusion reduction in the left inferior frontal gyrus (0.38 ± 0.34 vs. 0.77 ± 0.19, *p* < 0.001) (see Figure 6, anatomical location and description as shown in Table 3). No significant areas of high perfusion were observed in POCD patients in other brain regions, and the differences in perfusion in other brain regions were not statistically significant.

### 3.3. VBM Analysis of Cortical Grey Matter Based on 3D-T1WI

**Figure 7:**
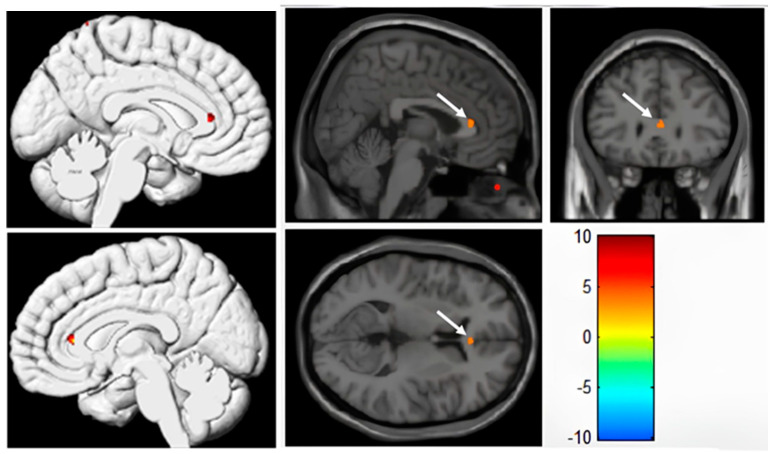
Differences in grey matter volume between the POCD and NPOCD groups. Voxel-wise comparison of the partial volume-corrected 3D-T1 revealed the volume of the right anterior cingulate gyrus (ACC) in patients with postoperative cognitive dysfunction (POCD) is slightly larger than that in patients without postoperative cognitive dysfunction (NPOCD). The colour gradient from blue to red represents varying levels of blood flow, with blue indicating low flow and red indicating high flow.

Among the 51 patients, a total of 51 sets of high-resolution thin-layer T1-weighted structural images were collected, with 9 sets belonging to POCD patients and 42 sets belonging to non-POCD patients. A two-sample *t*-test comparing cortical grey matter volume differences between the two groups of subjects showed statistical significance. The brain regions with significantly increased grey matter volume were mainly located in the right anterior cingulate gyrus. According to Brodmann Area (BA) partitioning, the regions with increased grey matter volume were mainly in BA24 (right anterior cingulate gyrus), which included 11 voxels. Compared to the non-POCD group, patients in the POCD group had a slightly larger cortical grey matter volume in the right anterior cingulate gyrus (0.23 ± 0.05 vs. 0.17 ± 0.03, *p* < 0.001) (see Figure 7, anatomical location and description as shown in Table 4). No significant cortical grey matter atrophy was observed in other brain regions of the POCD patients, and the differences in cortical grey matter volume in the other brain regions were not statistically significant.

### 3.4. SBM Analysis of Cortical Grey Matter Based on 3D-T1WI

Among the 51 sets of high-resolution thin-layer T1-weighted structural images collected, the surface maps of “Sulcus depth”, “Fractal dimension” and “Thickness” of every patient were evaluated. We found no significant association between global or regional “Sulcus depth”, “Fractal dimension” and “Thickness” and POCD incidence. This absence of association was robust with the inclusion of other covariates, such as age and years of education.

### 3.5. Predictive Performance of Single Parameter and Combination

**Figure 8:**
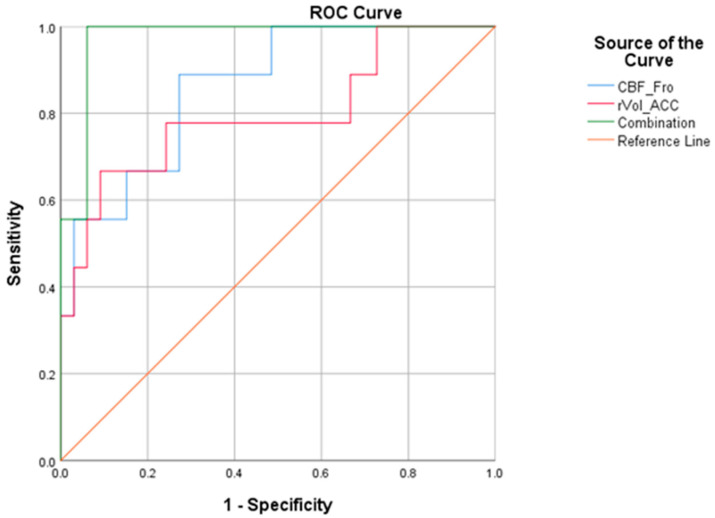
Single parameters and combination. CBF_Fro: CBF in the left inferior frontal gyrus; rVol_ACC: the normalised volume ratio of right anterior cingulate gyrus; Combination: combination of the two parameters.

As is shown in Figure 8, ROC curves were created and their AUC values were calculated, which is based on the individual and combined data of the left inferior frontal gyrus CBF and anterior cingulate cortex (ACC) volume. The AUC for the left inferior frontal gyrus CBF was 0.862, and for ACC volume, it was 0.798. However, the combined AUC for the left inferior frontal gyrus CBF and ACC volume was the highest at 0.973.

The cutoff values, sensitivity, specificity, Youden index and AUC for the individual parameter models are shown in Table 5: for the left inferior frontal gyrus CBF value (CBF_Fro): sensitivity of 0.889 and specificity of 0.727; for the anterior cingulate cortex volume (rVol_ACC): sensitivity of 0.667 and specificity of 0.909. The combined AUC for CBF_Fro and rVol_ACC is 0.973, demonstrating high sensitivity (1.0) and specificity (0.939).

## 4. Discussion

POCD manifests subtly in various ways, including memory decline, attention and concentration issues and language and communication difficulties [51]. Structural MRI scans are now routine, and ASL, a non-invasive and low-cost examination, provides an effective method for assessing early cognitive decline while only extending the scanning session by a few minutes. This study utilises MRI (structural and ASL) to predict POCD. While MRI is relatively expensive, the early identification of high-risk patients could potentially reduce long-term healthcare costs associated with POCD. The combined use of cortical grey matter volumetry and ASL-MRI showed superior predictive performance (AUC 0.973) compared to single modalities. This suggests a potential for more accurate patient stratification, which could improve resource allocation. The study focused on patients ≥60 years; given the higher risk of POCD in older adults, maintaining this age criterion seemed appropriate. The inclusion of patients undergoing laparoscopic or open abdominal surgery lasting >2 h could be a good selection criterion, as longer surgeries may increase POCD risk. We believe in the future, the time for MRI exams for high-risk patients will be shortened with the development of the technology.

Our research results showed that POCD patients had fewer years of education and lower IADL scores. Lower educational level is a known risk factor for POCD, suggesting that these patients had a reduced cognitive reserve (CR) [4]. Baseline cognitive impairment was also an important risk factor for POCD. Two studies about orthopaedic surgery and coronary angiography, respectively, highlighted that the existence of pre-existing cognitive impairment independently predicted the occurrence of POCD three months following the procedures [1,52]. POCD patients had higher Pittsburgh Sleep Quality Index scores. Poor sleep quality could be another screening criterion.

The cognitive function tests in Table 2 show several significant differences between POCD and non-POCD groups, particularly in postoperative assessments. These cognitive assessment results indicated that POCD patients primarily exhibited impairments in language and executive functions, which is consistent with clinical manifestations reported in the literature [50]. Mini-Mental State Examination (MMSE) scores were significantly lower in the POCD group (26 vs. 28, *p* = 0.015). A two-point difference, while statistically significant, may not always be clinically meaningful. However, it suggests a general decline in global cognitive function in POCD patients. Both the preoperative (5 vs. 10, *p* = 0.019) and postoperative (3 vs. 9.5, *p* < 0.001) scores of the Verbal Fluency Test (VFT) were significantly lower in the POCD group. The substantial difference suggests POCD patients have marked difficulties in word retrieval and executive function, which could significantly impact daily communication and decision-making abilities. The postoperative scores of TMT B were significantly different (300 vs. 272.5, *p* < 0.001). The marked difference suggests POCD patients have significant impairment in executive function, which could affect their ability to plan, organise and adapt to new situations. The pattern of deficits, particularly in VFT and TMT B, indicates that POCD in this cohort primarily affects executive function and language abilities. These impairments could have substantial impacts on patients’ daily functioning, decision-making capacity and quality of life. The results underscore the importance of comprehensive postoperative cognitive assessment and the need for targeted interventions to address these specific cognitive domains in POCD patients.

A large body of research indicates that reduced CBF is a sensitive marker of early cognitive impairment [53]. While the link with POCD remains unclear, a kind of hypothesis suggests that blood circulation disorders, including microcirculation disorders and insufficient cerebral blood flow, may occur during the surgical process, which may lead to inadequate blood supply to the brain and affect cognitive function [7]. In our study, we found that POCD subjects without pre-exiting cognitive dysfunction exhibited decreased cerebral blood flow in the left inferior frontal gyrus comparing with NPOCD. According to the results of the cognitive assessment in this experiment, the impairment of cognition in POCD patients was located in language and executive function, which is roughly similar to a previous report [51]. Those two functions are closely related to the prefrontal lobe [54].

Sena et al. found that Parkinson’s disease (PD) patients with dementia also showed a hypoperfusion in the left inferior frontal gyrus (IFG) compared with PD patients with normal cognition [55]. IFG is a part of the frontoparietal network (FPN), and the activity of the FPN is associated with advanced cognitive function [56]. Wang et al. also found that the CBF is reduced in the bilateral IFG of PD patients with mild cognitive impairment during a 2-year follow-up, and the reduced CBF is strongly related to executive function [57]. This hypoperfusion can lead to cognitive impairment partly due to the neurovascular unit dysfunction, disrupted neurotransmitter circuits and mitochondrial energy deficiency [58]. However, Xue et al. found a hyperperfusion in the left inferior frontal gyrus of POCD patients, which is opposite to ours [30]. The possible reason is that the patients in our cohort were older and less educated than Xue’s patients, which could lead to more cerebrovascular burden and less cognitive reserve (CR).

Secondly, the cortical grey matter volume in the right anterior cingulate cortex (ACC) of POCD is slightly bigger than that of NPOCD. The result probably is a compensatory manifestation. The ACC has very close connections with adjacent brain regions relating to emotions, cognition and executive control and motor control with prominent projections [59,60]. From our research results, it is evident that the patients in the POCD group show significant declines in language ability and executive function postoperatively, which may be related to reduced cerebral blood flow perfusion in the right inferior frontal triangle area. However, the Trail Making Test Part B (TMT B) scores of preoperative POCD patients, which provide a measure of cognitive function associated with the sequencing and set-shifting, did not show a significant decrease. Therefore, the larger volume of grey matter in the ACC cortex of POCD patients may be a compensatory effect against the reduced cerebral blood flow in the right inferior frontal triangle area. In fact, the result of Wang’s study also showed that a negative correlation between the CBF of the right ACC and the TMT B score [57].

Thirdly, in contrast to previous studies that focused on a single medical imaging technique, this study evaluated the diagnostic performance of single parameters and combinations. The AUC for ASL-MRI was 0.862 and the AUC for the right ACC normalised volume was 0.798. From the results, it can be observed that the diagnostic performance of ASL-MRI was superior to cortical grey matter volume. Additionally, this study found that the combination of MRI cortical GM volume and ASL-MRI improved diagnostic efficacy compared to single parameters (AUC of 0.973). Based on positron emission tomography (PET) and structural-MRI technologies, Takashi et al. found that the AUC for the combined application of PET and MRI (AUC = 0.985) was bigger than that of a single technology [61]. Compared to single-modality magnetic resonance imaging (MRI) techniques, the combination of multiple MRI modalities can significantly enhance the diagnostic and predictive efficacy of imaging techniques for neurodegenerative diseases.

Finally, based on the results of the SBM analysis, no significant statistical differences were found between the two groups of patients in terms of cortical thickness, depth of brain sulci and complexity of cortical structure. VBM provides a perspective on local differences in brain structure, allowing for the identification of changes in specific regions, while SBM offers a more comprehensive view of structural variations, enabling the recognition of changes in overall structural patterns, including correlations between different regions. Possible reasons to consider are the following: (1) Data quality has a significant impact on the results of SBM. If the data quality is poor or contains noise, SBM may not accurately identify structural pattern changes. VBM has relatively lower requirements for data quality as it mainly focuses on local volume and density differences. (2) The characteristics and quantity of the samples also affect the results of VBM and SBM. If there are specific types of structural differences in the samples, VBM may be more likely to detect these differences. However, SBM may require a larger sample size and more structural variations to accurately identify overall structural patterns.

This study has several limitations. Firstly, it is a single-centre study with a small sample size, which may affect the statistical power of the results; thus, bigger and multicentre studies should be performed to prove our results in the future. Secondly, although this project is a longitudinal study, data collection is still in progress, and currently only the results of preoperative and one-month postoperative assessments have been analysed. We have excluded the patients with pre-existing cognitive impairment diseases according to medical records and the baseline cognitive performance, as well as patients with massive cerebral infarctions and cerebral tumours according to the MR imaging. But, the discrimination between POCD and other cognitive impairment diseases as a real challenge still needs further experiments to prove. Moreover, from a single preoperative MRI examination, it is hard to establish a causal relationship between brain structural/functional changes and POCD; we need one or more postoperative MR images in order to observe the dynamic changes and decouple the surgical effects and other confounding factors; combine them with multi-dimensional data, such as clinical, imaging and molecular biology, to construct a more comprehensive pathogenesis model of POCD; or use twin control or propensity score matching to control the confounding factors.

The 20% definition actually originates from an old study, but it is still the widely accepted standard, despite some controversy; therefore, this criterion need to be updated and optimised, such as replacing it with the z score or the one standard deviation (1SD) definition. Additionally, the study only collected ASL-MRI data and clinical-scale information from the participants, lacking auxiliary diagnostics such as cerebrospinal fluid and other biomarkers. Fourth, this study is a single-centre study that used three different brands of MRI scanners for scanning. Although the scanning parameters were standardised, there may be subtle differences in parameters between different brands of machines, which could affect the accuracy of the research results to some extent. Also, the study did not fully correct for potential confounding factors, such as a history of diabetes, smoking habits and caffeine intake, and in order to further correct for potential confounding factors, demographic data with significant statistical differences were added as covariates for correction when performing the voxel-wise two-sided *t*-test. Lastly, in order to control the influence of various intraoperative confounding factors, future studies study should try to ensure that the amount and the duration of anaesthesia are roughly the same. However, some intraoperative factors may still affect the results of postoperative cognitive assessment, such as blood loss, intraoperative blood pressure and blood oxygen fluctuations. These data should be collected at the same time and corrected as covariates in future studies.

Based on the results of this study, it is possible to diagnose the high risk of POCD with MRI before surgery. Preoperative MRI examination should include functional and structural images. Before the examination, care should be taken to ensure that the patient is quiet and comfortable, and family members should accompany them if necessary. For these elderly patients, the anaesthesiologists should pay attention to the preoperative cognitive function screening and assessment and the surgeons need to perform a comprehensive preoperative health assessment and preoperatively communicate. As much as possible, anaesthetic drugs and methods with minimal effect on cognitive function should be selected. Then, a quiet, safe and familiar recovery environment should be provided to reduce postoperative confusion and anxiety. Postoperative cognitive function should be closely monitored, and regular follow-up and evaluation should be carried out to find and deal with changes in cognitive function over time. If it is necessary, medication can be given, such as attenuating the inflammatory response with dexamethasone.

## 5. Conclusions

The results of current study showed a difference in CBF at the left inferior frontal gyrus and grey matter at the right anterior cingulate gyrus between the two groups, and combined MRI cortical grey matter volumetry and ASL CBF was superior to either single measure in predicting early POCD. That means that we can find the high risk of POCD with preoperative MRI results for early prediction and prevention.

Future research recommendations include the following: (1) validation of the results in a multicentre, large-sample study; (2) exploration of multimodal prediction models for POCD by combining blood or CSF biomarkers; (3) conduction of a long-term follow-up study to observe the changes in brain structure and function in POCD patients.

## Figures and Tables

**Table 1:** Demographics.

	POCD (*n* = 9)	NPOCD (*n* = 42)	*p* Value
Age (year, median, IQR)	72 (64–78)	72 (66–77)	0.605 ^a^
Sex(male, %)	6, 66.67%	30, 71.43%	0.778 ^c^
Smoke (%)	3, 33.33%	11, 26.19%	0.981 ^c^
Alcohol (%)	4, 44.44%	12, 28.57%	0.592 ^c^
Hypertension (%)	8, 88.89%	21, 50%	0.077 ^c^
Diabetes (%)	3, 33.33%	4, 9.52%	0.166 ^c^
Anaesthesia (%)	3, 33.33%	11, 26.19%	0.981 ^c^
Education (year, median, IQR)	6 (2–6)	6 (6–9)	0.008 ^b^*
Exercise (min/d, median, IQR)	90 (30–240)	25 (0–67.50)	0.011 ^b^*
IADL (median, IQR)	0 (0–1)	0 (0–0)	0.024 ^b^*
PSQI (median, IQR)	7 (5–8)	3 (0–6)	0.040 ^b^*

IQR: Interquartile range, SD: standard deviation, IADL: Instrumental Activities of Daily Living, PSQI: Pittsburgh Sleep Quality Index, * *p* < 0.05, ^a^: Independent Samples *t*-test, ^b^: Mann–Whitney U test, ^c^: Chi-square test.

**Table 2:** Summary statistics of patients with and without POCD from different cognitive function tests.

	POCD (*n* = 9)	NPOCD (*n* = 42)	*p* Value
MMSE			
preoperative, median (IQR)	23.5 (20–26)	28 (27–29)	0.13 ^b^
postoperative, median (IQR)	26 (20–27)	28 (26.75–29)	0.015 ^b^*
difference, median (IQR)	0 (0–3.5)	0 (0–0.25)	0.12 ^b^
CDT			
preoperative, median (IQR)	1 (1–1)	1 (0–3)	0.698 ^b^
postoperative, median (IQR)	1 (0–1.5)	1 (0–3)	0.419 ^b^
difference, median (IQR)	0 (0–1)	0 (0–0)	0.558 ^b^
VFT			
preoperative, median (IQR)	5 (1–10)	10 (6–11)	0.019 ^b^*
postoperative, median (IQR)	3 (0–6.5)	9.5 (7.5–12)	<0.001 ^a#^
difference, median (IQR)	0 (0–2)	0 (−1–1)	0.022 ^b^*
TMT A			
preoperative, median (IQR)	97 (65–120)	101 (76.5–120)	0.334 ^b^
postoperative, median (IQR)	120 (94.5–144.5)	101.5 (77.5–120)	0.085 ^b^
difference, median (IQR)	−14 [(−57.5)–(−0.5)]	0 (−1–1.25)	0.08 ^b^
TMT B			
preoperative, median (IQR)	300 (128–300)	287.5 (170–300)	0.729 ^b^
postoperative, median (IQR)	300 (300–300)	272.5 (165–300)	<0.001 ^b#^
difference, median (IQR)	0 (−156–0)	0 (−2–1.25)	0.043 ^b^*
DST			
preoperative, median (IQR)	11 (9–11.5)	10 (8–12)	0.773 ^a^
postoperative, median (IQR)	10 (7.5–11)	10 (8–12)	0.588 ^a^
difference, median (IQR)	0 (0–2)	0 (−1–1)	0.106 ^b^
Stroop test			
preoperative, median (IQR)	11 (7.5–18)	15 (10–18.25)	0.18 ^a^
postoperative, median (IQR)	9 (6–19)	15 (10–18.25)	0.186 ^a^
difference, median (IQR)	0 (0–1)	0 (0–1)	0.989 ^b^

MMSE: Mini-Mental State Examination, CDT: Clock-Drawing Test, VFT: Verbal Fluency Test, TMT A and B: Trail Making Test A and B, DST: Forward and Backward Digit Span Test, Stroop: Stroop Colour and Word Test, IQR: interquartile range, SD: standard deviation, * *p* < 0.05, ^#^ *p* < 0.01, ^a^: Independent Samples *t*-test, ^b^: Mann–Whitney U test.

**Table 3:** Areas of different perfusion in the POCD compared to the NPOCD group.

Brain Area	Peak MNI Coordinate			Cluster Size	Statistical Value (T)
	x	y	z		
Left inferior frontal gyrus	−52	46	16	14	−4.3251

**Table 4:** Areas of different GM volume in the POCD compared to the NPOCD group.

Brain Area	Peak MNI Coordinate			Cluster Size	Statistical Value (T)
	x	y	z		
Right anterior cingulate gyrus	3	30	9	11	3.5365

**Table 5:** Sensitivity, specificity, cut-off values (based on Youden index) and AUC for single and combined parameter models.

	Cut-Off	Sensitivity	Specificity	Youden Index	AUC	
					Mean	95%CI
CBF_Fro	0.17	0.889	0.727	0.616	0.862	0.735, 0.988
rVol_ACC	0.29	0.667	0.909	0.576	0.798	0.608, 0.988
Combination	0.23	1	0.939	0.939	0.973	0.930, 1.000

## Data Availability

The raw data supporting the conclusions of this article will be made available by the authors on request.
